# Modeling methods of different tumor organoids and their application in tumor drug resistance research

**DOI:** 10.20517/cdr.2025.34

**Published:** 2025-07-01

**Authors:** Chengming Yang, Lushan Yang, Yuchen Feng, Xingyi Song, Shu Bai, Sheng Zhang, Mingjuan Sun

**Affiliations:** ^1^Department of Student Team, College of Basic Medical Sciences, Naval Medical University, Shanghai 200433, China.; ^2^Medical Oncology, Shanghai Cancer Center, Fudan University, Shanghai 200032, China.; ^3^Department of Biochemistry and Molecular Biology, Naval Medical University, Shanghai 200433, China.

**Keywords:** Cancer, tumor organoids, modeling, drug resistance, drug screening

## Abstract

Tumor organoids were modeled *in vitro* to mimic *in vivo* culture conditions, allowing tumor-derived tissue cells or isolated and purified tumor stem cells to self-assemble into 3D preclinical models that are similar to tissues and organs *in vivo*. Compared with traditional models, tumor organoids not only resemble parental tumors in histology and genomics, capturing their heterogeneity and drug response, but also provide an efficient platform for long-term culture, maintaining genetic stability and enabling gene manipulation. Therefore, tumor organoids have unique advantages in cancer drug resistance research. The paper covers: (1) Modeling methods of epithelial and non-epithelial tumor organoids, with special emphasis on the modeling of drug-resistant organoids; (2) Their use in drug resistance research, split into i. Therapeutic exploration (drug testing and screening) and ii. Mechanism investigation (use drug-resistant organoids to study drug resistance), including methods and findings from various teams.

## INTRODUCTION

Malignant tumor is one of the most common chronic diseases and one of the leading causes of death worldwide. According to the International Agency for Research on Cancer (IARC), nearly 20 million new cancer cases were diagnosed in 2022, resulting in approximately 9.7 million deaths. By 2050, the number of new cases worldwide is predicted to reach 35 million^[1]^. As more research has been dedicated to finding the right medication and therapy in the past three decades, the five-year survival rate of cancer patients has been greatly improved^[1]^. However, it is inevitable that most cancer patients are prone to develop drug resistance even under the corresponding cancer treatment^[2]^.

Cancer drug resistance studies, drug development, and treatment planning rely on accurate, consistent, and efficient preclinical models to mimic cancer cell behavior, including resistance. Traditional experimental models include 2D tumor cell lines, patient-derived xenograft (PDX), cell line-derived xenograft (CDX), and 3D cell spheres.

2D cultured cell lines offer distinct advantages, including low cost, high reproducibility, and compatibility with high-throughput drug screening. Their simplicity and scalability enable rapid mechanistic studies of tumor cell behavior at the molecular level. However, compared with *in vivo* cancer cells, 2D cultured cells have flat morphology and altered signaling networks, which fail to form multicellular resistance. They also lack the interactions between tumor microenvironment (TME) and cancer cells, leading to a loss of tumor structure and function *in vivo*. At the same time, primary tumors’ characteristics can be altered by long-term culture, reducing clinical relevance^[3]^.

PDX models retain key advantages such as preserving tumor heterogeneity and providing an *in vivo* platform for studying tumor-stroma interactions. However, PDX models face issues such as low transplantation success rates, long experimental cycles, high costs, and genetic manipulation challenges^[4]^. It is not suitable for the study of tumors with low malignancy or chronic progression. Moreover, due to the lack of certain immune cells in the treated tumor tissue, the model constructed in immunodeficient mice is not suitable for the evaluation of some immunotherapy drugs^[3]^.

In 2009, Sato *et al*. first created organoids from mouse intestinal stem cells^[5]^. This breakthrough was subsequently extended to tumor modeling. In 2011, they developed colorectal cancer organoids from patient tumor tissues^[6]^, marking the beginning of organoid applications in tumor modeling. Since its introduction, tumor organoids have been rapidly and widely utilized in various experimental platforms as a semi-*in vivo* model. These three-dimensional structures, formed through the self-assembly of tumor-derived tissue cells or purified cancer stem cells, can serve as personalized *in vitro* research models highly resembling real tumor tissues *in vivo*^[7]^. Tumor organoids can include immune cells^[8]^, tumor-associated fibroblasts, and other components^[9]^ when co-culturing with them, simulating aspects of the TME for drug evaluation. Meanwhile, tumor organoids offer a 3D structure similar to *in vivo* tumor tissue, maintaining cell polarity and matrix interactions, and supporting physical and chemical gradients. Therefore, tumor organoids do not have to adapt to the new environment, and can avoid genetic drift so as to be genetically stable after long-term passage.

Taken together, tumor organoids closely replicate the morphology and genomics of the original tumor, accurately reflecting its heterogeneity and drug sensitivity. This enhances drug development and screening accuracy^[10,11]^, overcoming limitations in preclinical models regarding tumor differentiation and drug response. Compared to PDX models, tumor organoids are more cost-effective and convenient, offering rapid model establishment, high culture efficiency, and an overall high success rate^[12]^. Additionally, organoids are amenable to cryopreservation and gene editing^[13-15]^, and avoid ethical issues, making them promising for cancer drug development and resistance research^[16,17]^, ultimately facilitating the transition from lab to clinic.

## METHODS OF MODELING DIFFERENT TUMOR ORGANOIDS

### Normal tumor organoid modeling methods

#### Basic operation steps

**Figure 1:**
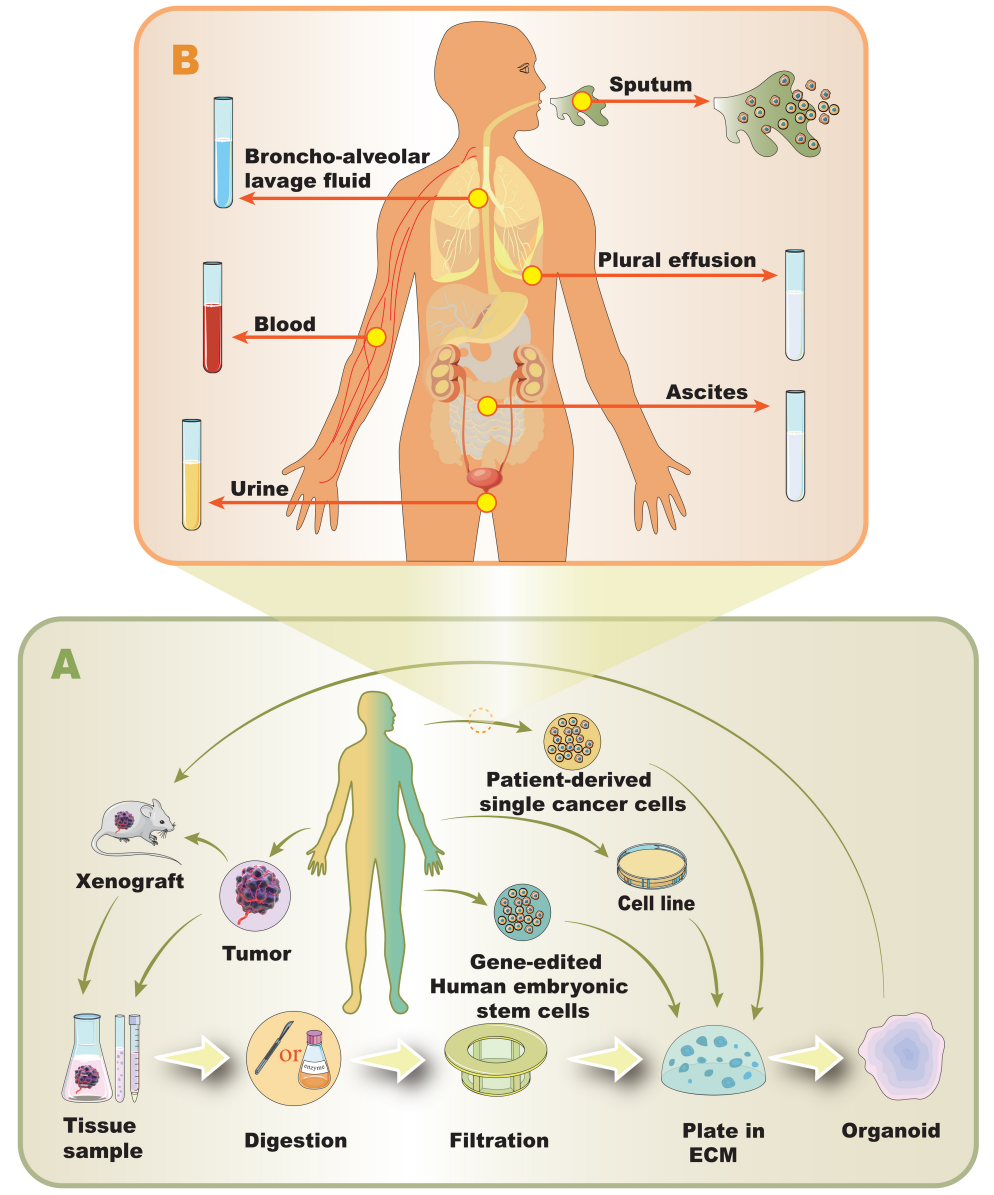
Overview of the organoid generation process: (A) Different modeling procedures of non-resistant tumor organoids: Tissue samples from xenografts or tumors are collected and digested to isolate single cells. These cells are then filtered and plated in an ECM to culture organoids. Alternatively, organoids can be generated from patient-derived single cancer cells, hESCs, or cell lines;(B) Schematic representation of the non-surgical sampling methods: Organoids can be derived from various body fluids and tissues, including bronchoalveolar lavage fluid, sputum, pleural effusion, ascites, blood, and urine, which are collected from different parts of the human body. ECM: Extracellular matrix.

As tumor organoid modeling research progresses, various schemes have been explored^[18-22]^. Despite differences, most of them share a core set of common steps [Figure 1A]. Below is a streamlined overview of the primary culture process^[23,24]^.

(1) Sampling: Obtain required tumor samples by suitable means. (2) Cell Mass Preparation: Select the appropriate size of cell mass via mechanical disruption (pipetting), enzymatic digestion, filtration, and centrifugation. Some steps may be skipped based on the sample source (3) Density adjustment: Resuspend the pellet in the working medium, and determine cell density to calculate medium and extracellular matrix (ECM) needs. Adjust cell density by dilution or post-centrifugation resuspension. (4) ECM Mixing and Plating: Combine pellets with ECM or resuspend in medium-ECM mix. Place drops in pre-warmed wells, and incubate at 37 °C, 5% CO_2_, to solidify ECM. After solidification, add pre-warmed organoid medium to each well.

For detailed procedures, including preparation, tips, and steps for primary culture, passaging, cryopreservation, and thawing of organoids, refer to Supplementary Materials.

#### Specific operating procedures

Due to the heterogeneity of tumors, tumors of different sites or tumors of the same site from different patients have different modeling complexity and unique requirements for modeling conditions. As a result, different tumors often have a variety of modeling methods; even for the same tumor, the protocols of different research teams may be different in details.

Sampling Organoids can be derived through either surgical or non-surgical methods. Surgical approaches include the acquisition of esophageal and oral tissue samples via endoscopic biopsy^[25]^, and urethral epithelial cancer samples obtained from urethral resection specimens^[24,26,27]^. Non-surgical sources include bladder cancer cells isolated from urine^[28]^; non-small-cell lung cancer cells extracted from pleural effusions, bronchoalveolar lavage, or sputum^[29]^; circulating tumor stem cells collected from peripheral blood^[29,30]^; and ovarian cancer cells obtained from ascitic fluid^[31]^ [Figure 1B]. Organoids can also be established from non-human sources such as PDX^[32]^, established cell lines^[33]^, or murine sources^[34]^. Additionally, gene-edited human embryonic stem cells (hESCs) serve as a valuable source for organoid generation^[35,36]^. All research involving human samples - including peripheral blood, ascites, and tissue biopsies - must comply with applicable institutional and governmental ethical regulations, and consent must be obtained from all participants prior to the sample collection.

*In vitro* culture After obtaining the sample, remove all non-epithelial tissue (e.g., muscle, fat) with tweezers and surgical scissors or scalpels, then cut the primary tumor tissues into 1-3 mm^3^ pieces. The tissues are then digested and monitored with collagenase/hyaluronidase and TrypLE Express enzymes as appropriate for the tumor type. For incubation < 2 h, the mixed tissue contents are agitated every 10-15 min by vigorous shaking and pipetting with P1000 pipettes. For overnight incubations, place the mixture on a shaker and add 10 µM of ROCK inhibitor during digestion to improve the growth efficiency. Based on experience, digestion is considered completed when clusters of 2-10 cells become visible, and these can be further dissociated by gentle pipetting. Cell strains are then passed through to screen for appropriately sized single cell or cell clusters. The digestion time and filter pore size (70 µm/100 µm) are determined according to the tumor type and the specific situation. When starting to culture a new type of tissue, it is recommended to take small samples from the digested fluid during the digestion process and plate them to determine the optimal digestion time for the tissue. Compared to the surgical route, the non-surgical approaches and non-human sources do not require traumatic surgery and have less damage to the human body^[20,30,37]^. In addition, some non-surgical approaches and cell line-derived primary modeling do not require collagenase/hyaluronidase and TrypLE Express enzymes for digestion or filtration^[29,33,37]^, making the procedures relatively simple. However, bronchoalveolar lavage fluid still requires filtration^[10]^, and blood-derived circulating tumors require additional purification steps^[20]^. After obtaining the desired cells or cell clusters, dilute them as needed and mix them in three-dimensional ECM hydrogel, which includes basement membrane extracts (BME), Matrigel, and Geltrex^[38]^, with different choices for the different teams. During the dispensing step, some protocols involve plating the cell-ECM mixture into 98/48/24-well plates, typically using 10-20 μL per drop to form hemispherical 3D structures at the bottom of each well. The plates are then inverted to prevent the cells from setting and adhering to the bottom surface, followed by incubation at 37 °C with 5% CO2 for 15-30 min to allow the ECM to solidify. This method is often used when the available number of tumor cells is relatively low, such as in samples derived from bladder cancer cells from urine^[28]^ or bone metastatic prostate cancer tissue from PDX. As a result, smaller amounts of ECM are also typically used. However, some protocols apply the same method even when cell numbers are sufficient^[24,26]^. In some schemes with a large number of cells, the cell-ECM suspension was directly dropped in the middle of the plate (6-well plate or plate with a larger pore size) precoated with the mixture of medium and ECM to prevent cells from adhering to the bottom of the plate^[39]^. In this method, researchers do not need to turn over the plate and the droplets were cured at 37 °C and 5% CO2 for 30 min, but more cell-ECM suspension is required per well. After dispensing, culture medium containing a mixture of growth factors was added to each well. The key components of the mixture usually include: activators of Wnt signal; ligands for tyrosine receptor kinases, such as epidermal growth factor (EGF), which promotes epithelial cell proliferation^[40]^; inhibitors of the transforming growth factor-β/bone morphogenetic protein signaling pathway, such as Noggin, which is known to induce epithelial differentiation. The components of different tumor organoid culture media are diverse. Additionally, specific parameters such as centrifugation speed and duration, the volume of cell-ECM suspension dispensed per well, the splitting ratio during passaging, and the method of isolating organoids from the ECM during modeling differ among protocols. The detailed differences in primary culture procedures for organoid modeling across various tumor types are summarized in Table 1. Once tumor organoids are established, xenograft-derived organoids can subsequently be generated through orthotopic grafting of the organoids^[39]^.

**Table 1:** Differences in organoid modeling for different types of tumors

**Tumor**		**Bladder cancer**					**Gallbladder cancer**	**Renal pelvis cancer**	**Lung cancer**	**Ovarian cancer**	**Esophageal and haryngeal squamous cell carcinoma**	**Papillary thyroid carcinoma**	**Rhabdomyosarcoma**	**Glioblastoma**
Reference		[28]	[26]		[24]	[39]	[41]	[26]	[42]	[43]	[33]	[44]	[45]	[46]
Year of publication		2023	2022		2022	2020	2018	2022	2022	2023	2020	2023	2022	2021
Organoid line name		-	BCO#140	BCO#147	-	SCBO-1-6	-	BCO#154	-	-	-	-	RMS.*?	-
Survival time		-	28 passages	2 h	> 7d	-	> 6 months	20 passages	-	-	2-3 weeks	> 6 months	> 6 months	-
Tumor sample count		35	-	-	-	23	-	-	114	-	-	27	46	53
Organoid formation count		29	-	-	-	16	-	-	162	-	-	22	19	70
Survival rate		83%	-	-	-	70%	-	-	76%	-	71.40%	81.50%	41%	91.40%
Source		Tissue	Tissue	Tissue	Tissue	Tissue	Tissue	Tissue	Lung effusions and tissues	Tissue	Tumors and adjacent mucosa	Tissue	Tissue	Tissue
Centrifugation speed		250 g	480 g	480 g	261 g	350 g	1,000 g	480 g	300 g	1,000 g	2,000 rpm	-	300 g	Gentle rotation
Centrifugation time		10 min	10 min	10 min	5 min	5 min	5 min	10 min	5 min	5 min	5 min	-	5 min	10 min
Filter pore size		-	70 µm	70 µm	100 μm/37 µm	100 µm	100 µm	70 µm	70 µm	70 µm	70 µm	70 μm	70 µm	-
ECM	BME	3X cell suspension	500 µL	500 µL	2x organoid medium	-	-	500 µL	0	-	0	-	2/3 volume	0
	Matrigel	0	30 μL	30 μL	0	-	-	30 μL	200 μL (2X cell suspension)	-	50 μL	-	0	0
	Geltrex	0	0	0	0	-	-	0	0	-	0	-	0	0
Collagenase/hyaluronidase	Incubation time	-	30 min	30 min	1-2 h	15 min	0	30 min	2 h	1 h	45 min	-	25 min	0
	Dosage	-	3,000 U/mL and 1,000U/mL	3,000 U/mL and 1,000U/mL	1 mL 10x	1:10 dilution	0	3,000 U/mL and 1,000 U/mL	1 mg/mL	-	5 mg/mL	-	-	0
TrypLE express enzymes	Incubation time	-	-	-	-	3 min	30-60 min	-	-	-	-	-	3-10 min	0
	Dosage	-	-	-	-	5 mL	2.5 mg/mL Collagenase IV	-	-	-	-	-	-	0
Cell density		2 × 10^6^ /mL	4 × 10^6^ /mL	4 × 10^6^ /mL	-	4 × 10^6^ /mL	-	4 × 10^6^ /mL	-	10,000/ 50 μL~20,000 cells/ 50 μL	4 × 10^5^ /mL	-	-	-
Cell-ECM suspension per well		20 μL	40 μL	40 μL	100 μL	250 μL	-	40 μL	300 μL	-	50 μL	-	5-10 μL	4 mL, no ECM
Well plates		48	24	24	96	6	6	24	6	-	24	-	24/48	6

The regular expression “RMS.*?” is used to concisely represent the names of multiple organoids in the table: “RMS” is the common prefix of all these organoid names. “.*?” is a regular expression pattern where: “.” represents any character (except for a newline). “*” means the preceding character (in this case, any character) can be matched zero or more times. “?” makes the match non - greedy, so it matches as few characters as possible to satisfy the pattern. By using “RMS.*?”, we can indicate all organoid names starting with “RMS” in a concise way, saving space in the table while clearly conveying the information. ECM: Extracellular matrix; BME: basement membrane extracts.

#### Modeling of non-epithelial origin

Due to their intrinsic self-organization capacity and clinical relevance, organoid technologies initially focused on epithelial tissues and later extended to related cancer types. Epithelial cells exhibit polarized structures and contain well-defined stem cell populations (e.g., LGR5+ intestinal stem cells^[5]^), enabling *in vitro* recapitulation of tissue architecture. Early breakthroughs, such as intestinal organoid models, demonstrated that epithelial stem cells could regenerate functional units (e.g., crypt-villus systems^[5]^) under controlled conditions. Furthermore, due to the scarcity of tissue samples from non-epithelial cancers, the application of this technology in the domain of non-epithelial cancers is relatively backward. Nevertheless, aside from organoid models derived from epithelial tissue, two types of non-epithelial tumors have been successfully developed.

Rhabdomyosarcoma Meister *et al*.’s study indeed demonstrated the applicability of organoid technology to tumors of mesenchymal origin, such as rhabdomyosarcoma (RMS)^[45]^, for drug screening and gene editing. To optimize RMS organoid cultivation, researchers have refined processes from sample collection to culture, using biopsies or resection specimens, and sometimes non-solid samples from bone marrow aspirations. Post-sampling, tissues are transferred to a collection medium to maintain viability, then minced with a scalpel under sterile conditions and overlaid with culture medium containing BME. Notably, the practice of adding BME before mincing is less common in the preparation of epithelial-origin tumor organoids. High red blood cell content samples are treated with red blood cell lysis buffer in bone marrow cases. Various medium formulations are systematically tested to find the optimal growth conditions for RMS organoids, including necessary growth factors and supplements, and the use of BME to aid cell attachment and growth. These optimization steps significantly enhance the quality and efficacy of organoid models.

Glioblastoma Glioblastoma (GBM) is even more challenging to model. Thus, Linkous *et al*. started with hESCs and induced pluripotent stem cells (iPSCs)^[47]^. Brain organoids gradually evolve through the stages of embryoid bodies and neural rosettes. Subsequently, they extracted tumor tissue from surgical samples of GBM patients and enzymatically isolated tumor cells into individual cells, which were then cultivated in neural basal medium (NBE) to sustain glioma stem cell (GSCs) properties^[47]^. Then, patient-derived GSCs were co-cultured with brain organoids to facilitate the interaction between GSCs and organoids. GSCs are capable of migrating, invading, and proliferating, eventually forming tumor models in brain organoids that structurally resemble those observed in GBM patients^[47]^.

Jacob *et al*. developed a method to rapidly create glioblastoma organoids (GBOs) from fresh tumor samples without single-cell isolation^[46]^. They used a serum-free medium without EGF/basic fibroblast growth factor (bFGF) or additional ECM. Tumor tissues were minced into small pieces 0.5 to 1 mm in diameter, treated with red blood cell lysis buffer, and incubated in GBO medium with rotational shaking. 3/4 of the medium was partially replaced every 48 h to promote organoid formation, with rounded organoids (with a spherical morphology) forming within 1-2 weeks. To prevent necrosis, older GBOs were cut into 200-500 μm pieces. This method preserves tumor heterogeneity and microenvironment features such as hypoxia and microvasculature, maintaining natural cell interactions without clonal selection biases.

#### Comparison of modeling methods

Several sampling methods can be categorized as human-derived or non-human-derived based on their source. Human-derived organoids, primarily obtained from patients, face challenges such as complex sampling procedures and low success rates. However, cultured patient-derived organoids (PDOs) enable personalized treatment strategies. In contrast, non-human-derived organoids are more convenient for scientific research, particularly in high-throughput drug screening and mechanistic studies, though they lack patient-specific features.

Human-derived sampling methods are further divided into surgical and non-surgical approaches. Surgical specimens (e.g., tumor resections) provide ample cellular material but involve invasive procedures and risk contamination from adjacent non-target tissues (e.g., adipose or stromal components). Non-surgical methods, such as endoscopic or needle biopsies, are less invasive but restricted to specific tumor types. Liquid biopsy, while minimally invasive, remains technically underdeveloped and yields limited sample volume, restricting its utility in organoid establishment.

The modeling approaches for tumor research vary significantly among 2D monolayers, 3D spheroids, and organoids. Conventional 2D monolayers, reliant on anchorage-dependent growth over rigid surfaces, prioritize scalability but fail to replicate physiological cell-cell/matrix interactions, compromising translational relevance. Self-assembled 3D spheroids partially restore tissue architecture through emergent hypoxia gradients and cell polarity but lack standardized ECM support and stromal heterogeneity. In contrast, organoids are engineered within ECM scaffolds (e.g., Matrigel) supplemented with niche-specific factors, recapitulating TME complexity. This biomimetic system sustains long-term proliferation, multilineage differentiation, and drug response patterns mirroring *in vivo* behavior, making organoids superior to 2D and 3D models for investigating mechanisms of tumor resistance. A comprehensive comparison of these methodologies is detailed in Table 2.

**Table 2:** Comparative analysis of different modeling methods

**Category**		**Sampling method**	**Samples**	**Procedure invasiveness**	**Limitations**	**Applications**	**Cultivation features**
Tumor organoids	Human-derived	Surgical methods^[28]^	Tumor resection	Invasive (but therapeutic)	Contamination from adjacent tissues	Provides abundant cellular material	ECM-supported, stromal co-culture
		Non-surgical methods^[48]^	Endoscopic biopsy, needle biopsy	Moderate^†^	Limited to specific tumor types	Local tumor diagnosis, small sample research	Requires minimal tissue input
		Liquid biopsy^[42]^	Blood/body fluid samples	Minimally invasive^†^	Technologically underdeveloped; limited volume	Non-invasive monitoring, early cancer screening	Low yield; requires CTC enrichment
	Non-human-derived^[33]^	-	Animal models/ cultured organoids	-	Lack of individualized features	Drug development, basic mechanism research	Standardized conditions, high reproducibility
2D culture^[49]^		-	Monolayer cells	-	Poor TME mimicry	Rapid screening, mechanistic assays	Rigid substrate, no ECM
3D spheres^[50]^		-	Cell aggregates	-	Limited ECM and stromal diversity	Hypoxia/drug penetration studies	Self-assembled, no controlled niche factors

^†^For organoid cultivation: additional sampling beyond diagnostic procedures may increase invasiveness. ECM: Extracellular matrix; TME: tumor microenvironmen

### Drug-resistant organoid modeling

Due to the phenomenon of drug resistance, most of the tumors in cancer patients tend to undergo changes in morphology, signaling pathways, gene expression, and other characteristics after a period of treatment. These adaptations allow tumors to survive in the new therapeutic environment, ultimately leading to the emergence of drug-resistant tumors^[2]^. As these drug-resistant tumors differ significantly from the original untreated tumors, studying their properties *in vitro* using organoid models requires more than just conventional tumor organoids. It is also essential to generate organoids derived from drug-resistant tumors.

**Figure 2:**
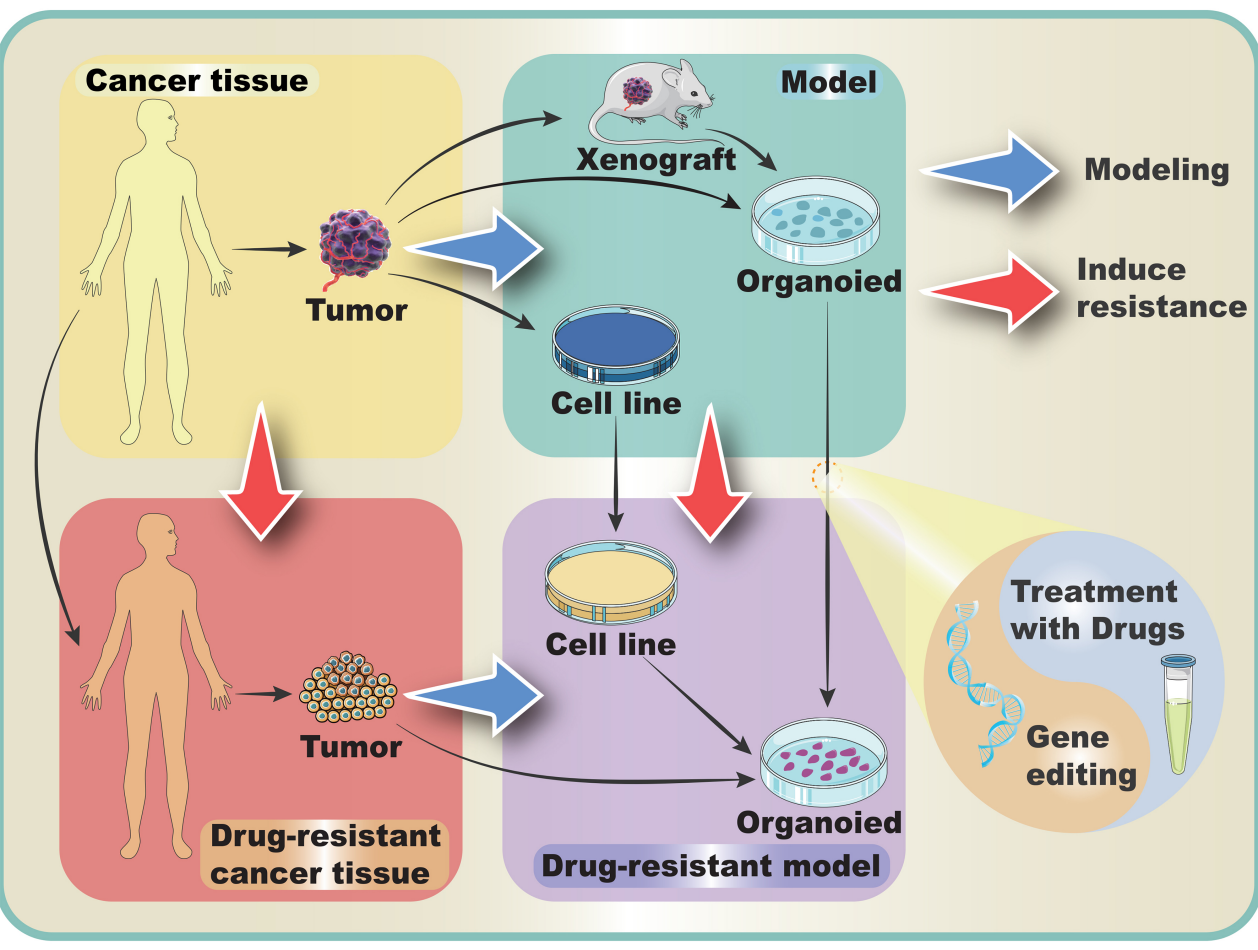
Different modeling procedures of drug-resistant tumor organoids: The blue pathway outlines the modeling process where tumor tissue is collected and used to create xenografts or cell lines, which are finally cultured into organoids for research. The red pathway details the induction of drug resistance. Drug-resistant tumor tissues are derived from patients with primary or secondary drug resistance. Normal cell lines and organoids can be transformed into drug-resistant cell lines and organoids through treatment with drugs or gene editing.

Currently, the modeling of drug-resistant tumor organoids is mainly developed from three aspects: directly generated from drug-resistant tumor tissues or cells^[33,51,52]^, induced establishment based on normal tumor organoids^[32,53]^, and modified by molecular biological techniques (e.g., gene editing)^[54,55]^ [Figure 2]. The third method is mainly used in the late stage of tumor drug resistance mechanism research to verify the proposed hypothesis of the drug resistance mechanism.

#### Based on drug-resistant tumor tissue

The method of generating drug-resistant tumor organoids based on drug-resistant tumor tissues or cells is largely consistent with that used for conventional tumor organoid modeling. Compared to the conventional approach - first establishing tumor organoids and then inducing drug resistance - this method eliminates the induction step, significantly reducing the time required and offering greater convenience and efficiency. Furthermore, organoids generated in this way harbor drug resistance mutations that closely resemble those found in human tumors, providing substantial advantages for research and therapeutic testing. Drug-resistant organoids can be derived from human drug-resistant tumor tissues^[43,51,52,56,57]^ or even from drug-resistant cell line models^[33]^. So far, there is no report on directly establishing drug-resistant organoids from drug-resistant PDX models.

#### IC_50_-guided induction

Since drug-resistant tumor tissue is not always available, as it is meaningless to perform surgery after some drug treatments cause drug resistance and there is no way or reason to obtain the tumor tissue, many programs choose to establish drug-resistant tumor organoids based on the induction of normal tumor organoids. For decades, scientists have employed IC_50_ (half-maximal inhibitory concentration) as a foundational metric to initiate drug resistance induction in preclinical models^[58]^. This traditional approach involves exposing cells or organoids to a drug concentration equivalent to the IC_50_, followed by incremental increases in dosage as tolerance develops, thereby mimicking the evolutionary pressure driving resistance *in vivo*. While this methodology has been widely adopted across cancer research, its application often requires empirical adjustments due to variability in tumor responsiveness and experimental conditions. Based on the variations in drug sensitivity across different models and changes post-drug induction, Yu *et al*. proposed a patented method for the standardized construction of drug-resistant tumor organoids^[59]^. They first assessed the drug sensitivity of the tumor organoids and determined the IC_50_ value, which was then used as the initial drug concentration for screening and expanding drug-resistant strains. A subset of the organoids was subsequently subjected to drug sensitivity testing, and the newly obtained IC_50_ value was used as the drug concentration for the next round of induction and amplification. Unlike arbitrarily set concentrations, this method employs IC_50_ as a dynamic reference for cyclic drug dosing to induce resistance *in vitro*, effectively addressing the challenge of selecting appropriate initial concentrations for different tumor organoids and thereby improving the success rate of induction.

#### Induction not based on IC_50_

In recent years, few tumor organoid drug-resistance modeling schemes have demonstrated a high degree of consistency with the method proposed in the referenced patent. Some protocols used the IC_50_ value only as an initial treatment concentration, adjusting the dose as needed. Others do not base the induction process on IC_50_ at all; instead, they predetermine the initial treatment concentration and either maintain it throughout the process or increase it depending on the response^[53,54,60]^. In some cases, the initial concentration is not even specified. Common d viability assays used in these protocols include WST-1, MTT, CCK-8, and CellTiterGlo3D, with CCK-8 being the most frequently used. It is speculated that the full effect of some drugs may take longer to manifest than the incubation time permitted by assays such as CCK-8. When IC_50_ is used as the treatment concentration, viable cell numbers may not meet expectations after a given period, or cell viability may be insufficient to sustain further expansion. Therefore, the induction conditions must be adapted to the specific experimental situation. For example, Harada *et al*. treated drug-sensitive tumor organoids with a range of concentrations and selected the organoids that tolerated the highest concentration for further passaging. In subsequent cycles, multiple concentrations were again tested, with the lowest concentration set as the highest concentration tolerated in the previous cycle^[54]^. This adaptive approach is suitable for inducing drug resistance in various tumor organoid models. By continuously approaching the maximum tolerated drug concentration in each cycle without the need for repeated drug sensitivity testing, this method enables highly efficient induction of drug resistance and serves as a valuable reference for researchers.

Details of sensitivity testing methods, which are crucial for accurately identifying drug resistance and guiding subsequent experimental design. Typically, a drug-resistant tumor organoid model is considered successfully established when the treatment concentration exceeds twice the parental IC_50_, and the IC_50_ of the resistant organoids increases to more than three times the original value^[54]^. In some studies, resistant organoids were identified without formal drug susceptibility testing. For instance, Lee *et al*. used an Alamar Blue activity assay to confirm resistance to APTD in organoid spheres. After four consecutive weeks of APDT treatment, the PDX organoids remained metabolically active, indicating the acquisition of drug resistance^[32]^. A summary of modeling methods for various drug-resistant tumor organoids is provided in Table 3.

**Table 3:** Modeling methods for various drug-resistant tumor organoids

**Method and principle**	**Advantages**	**Disadvantages**	**Reference**	**Year of publication**	**Tumor**	**Drug**	**Organoid line name**	**Original IC_50_^†^**	**Drug resistance IC_50_**	**Culture cycle**
Induction based on IC_50_: each generation concentration is the current IC_50_	Scientific induction, higher success rate	Time-consuming, needs adjustments	[59]	2023	Bladder cancer	Gemcitabine	-	5.89 μM	49.19 μM	5 rounds
					Bladder cancer	Cisplatin	-	2.45 μM	11.43 μM	6 rounds
					Colorectal cancer	Paclitaxel	-	0.38 μM	3.10 μM	7 rounds
					Lung cancer	5-FU	-	1.27 μM	14.12 μM	8 rounds
Induction not based on IC_50_: constant drug concentration treatment	Simple, no IC_50_ needed	Suboptimal induction	[32]	2022	Prostate carcinoma	Androgen pathway directed therapy	PCSD1	-	-	4 weeks
			[60]	2021	Pancreatic cancer	FOLFIRINOX	FoXR1	0.782 μM	0.295 μM	6 cycles, each time 72 h
Induction not based on IC_50_: single concentration increment	Efficient induction	Time-consuming	[53]	2020	Gastric cancer	5-FU	GCO1	2.9 μM	20.3 μM	118 days
							GCO2	5.3 μM	36.2 μM	118 days
							GCO3	6.9 μM	27.1 μM	160 days
							GCO4	4.6 μM	44.2 μM	120 days
Induction not based on IC_50_: multi-concentration, select the organoids that can tolerate the highest concentration	Efficient, no frequent testing	Time-consuming	[54]	2021	Gastric cancer	L-OHP	K1	9.6 μM	33.6 μM	50 days
							K24	34.7 μM	110.2 μM	50 days
							K31	5.6 μM	40.7 μM	50 days
Modeling based on human drug-resistant tumor tissue: directly from drug-resistant tissue	Accurate model, reflects human tumors	Tissue availability issue	[61]	2023	Pancreatic cancer	Gemcitabine + albumin paclitaxel	PAC-388	-	-	-
							PAC-352	-	-	-
			[43]	2020	Ovarian cancer	Cisplatin	-	-	-	-
Gene knockout/overexpression: genetic engineering for gene control	Precise gene manipulation	Complex operation, off-target risk	[55]	2023	Ovarian cancer	Gemcitabine	-	Positively correlated with PRKRA expression		-
			[62]	2022	Ovarian cancer	Sorafenib	-	Positively correlated with BBOX1-AS1 expression		-

^†^Original IC_50_ values reflect the inherent resistance of primary tumor organoids prior to induction.

## TUMOR DRUG RESISTANCE ORGANOID RESEARCH APPLICATION

### Drug resistance testing

Clinical guidelines often advise against surgery for advanced bladder cancer due to the risk of systemic metastasis, favoring radiotherapy and chemotherapy instead. However, drug resistance can emerge, making personalized treatment plans crucial. Using 3D cultured tumor cells to pre-screen drugs can reduce risks, improve survival rates, and enhance quality of life. Studies show that tumor organoids’ *in vitro* responses often align with patients’ therapeutic responses^[11]^, indicating their potential for predicting patient reactions to clinical treatments^[13,63]^. At the same time, the economic value of using organoid drug sensitivity testing technology for anticancer drug screening is also considerable. By constructing a decision tree model, Li *et al*. concluded that the use of tumor organoid drug sensitivity testing technology for drug guidance could reduce the drug cost of ovarian cancer patients by 7,413.07~16,384.97 yuan by improving the treatment efficiency and reducing the change of drugs^[64]^. To a certain extent, it can reduce the economic burden of patients.

Drug resistance testing, or drug sensitivity testing, in tumor organoids follows a process akin to that used for cell lines, with critical optimizations tailored to 3D architecture and microenvironment preservation. Batul *et al*. published an expert consensus on drug sensitivity testing in patient-derived tumor organoids^[65]^. Meanwhile, in recent studies, organoids are passaged, cultured to recover structural integrity, harvested through enzymatic digestion (e.g., TrypLE Express), filtered to remove debris, counted, and plated at the desired density (e.g., 1 × 10^4^ cells/well) in low-concentration hydrogels (2%-10% Matrigel) to balance rigidity and nutrient diffusion. To ensure consistency, pre-treatment recovery periods of 24-48 h are mandated to stabilize organoid polarity and cell-cell interactions before drug exposure. After exposure, metrics are used to measure drug sensitivity. Protocols diverge in details such as ECM composition, detection kit selection, organoid size and exposure windows. In terms of time interval, drug exposure readings range from 1 to 24 days, averaging 5 to 6 days, which is longer than typical cell line tests to accommodate delayed drug effects in 3D systems. For drugs like cetuximab exhibiting non-sigmoidal dose-response curves, IC_50_ calculation is challenging. Here, area-under-curve (AUC) analysis can be used to capture dynamic resistance phenotypes instead^[66]^.

In routine drug sensitivity testing with PDOs, variations in size, shape, and growth rate can affect experimental accuracy and reproducibility. Single-cell-derived tumor organoid^[67]^ technology can overcome these limitations^[63]^. A study comparing single-cell dissociated GI tumor organoids with standard-cultured organoids found that single cells were smaller, more uniform, and had similar drug sensitivities to their parent organoids^[63]^. This suggests that single-cell-derived organoids can enable rapid, high-throughput drug screening in GI tumors. Cui *et al*. introduced an advanced bioprinting strategy for tumor organoids, utilizing bioinks containing heterogeneous cancer cells to precisely replicate tumor complexity^[68]^. This 3D-printed organoid model mimics *in vivo* tumor architecture, enabling dynamic assessment of drug responses, including permeability and cytotoxicity. Notably, the bioprinted organoids preserved radial oxygen gradients and cellular interactions, enabling the evaluation of resistance mechanisms in diverse tumor subtypes.

### Drug screening

Drug screening^[66]^ involves testing various compounds to identify effective treatments, playing a critical role in drug development and the evaluation of disease heterogeneity. Herpers *et al*. leveraged patient-derived colorectal cancer organoids for high-throughput screening of over 500 bispecific antibodies^[69]^. Among these, they identified MCLA-158, an EGFR × LGR5 bispecific antibody, that selectively degraded EGFR in LGR5+ cancer stem cells while sparing healthy tissues. In recent years, advances in tech have improved organoid-based drug screening methodologies.

#### Data acquisition

Data acquisition in organoid studies presents several challenges, including time-consuming procedures and the need for re-culturing if initial experiments fail. Tran *et al*. addressed this by using EZSPHERE 12-well plates with microwells for 3D aggregate formation and mass culture of multi-cyst kidney organoids^[70]^. For screening purposes, methylcellulose plates were employed to embed the organoids and prevent their movement. However, this method requires manual cell selection under a microscope, which can be technically challenging. Norrie *et al*. applied targeted RNA sequencing to monitor gene expression and assess cell phenotypes within organoids^[35]^. This approach generates detailed molecular data that are often lacking in standard drug screening protocols, enabling more comprehensive analysis of complex phenotypes and drug mechanisms of action.

Wang *et al*. developed a technique to visualize lipid dynamics in organoids in real time following drug treatment^[71]^. By labeling PLIN2, a protein abundant in the liver and associated with lipid droplets, they tracked changes in lipid content over time. The use of fluorescently tagged endogenous PLIN2 enabled continuous monitoring of organoid fluorescence signals, offering insights into drug-induced alterations in lipid metabolism. This PLIN2-based reporter system provides a real-time platform for studying drug effects on steatosis but is limited to organoid models with high fat content and substantial PLIN2 expression, such as liver organoids.

#### Data processing

In terms of data analysis, Kong *et al*. proposed a machine learning framework for network-based analysis to use pharmacogenomic data in 3D organoid culture models to identify powerful drug biomarkers and predict drug response in cancer patients^[72]^, Zhao *et al*. developed a morphological screening method for analysis pipelines^[73]^. Park *et al*. shed light on the applicable direction and future development of the emerging organoid chip. The organoid chip is essentially a fluid control device at the micro and nano level, capable of simulating the actual distribution of morphogens *in vivo*, rather than the traditional uniform distribution, thereby making the organoid model more realistic^[9,74,75]^.

Microarrays allow for the quantification of biochemical indicators in microdomains, enabling more precise high-throughput data generation and analysis. Integrating lensless imaging systems onto chips provides real-time organoid imaging, offering clear visualization of drug screening processes and the ability to observe organoid changes post-drug administration. Microfluidic chips in drug screening can mimic capillaries, facilitating organoid integration, vascular network construction, and simulating *in vivo* drug delivery pathways. Advances in micro and nanotech allow these chips to simulate multi-organ interactions, creating a multi-organoid drug screening platform that reflects *in vivo* pharmacokinetics.

Chip technology can indeed integrate with other traditional or emerging technologies. Wu *et al*. combined superhydrophobic micropore array chip data with hybrid RNA-Seq methods for genome-wide RNA output analysis that aligns with phenotypic data, significantly reducing consumables loss and the cost of single RNA-Seq samples^[76]^. However, organoids grown on microfluidic chips may disrupt the ECM simulated by biomaterials due to their own stretching and high-throughput fluid perfusion, leading to unstable adhesion or leakage over time. Thus, there is a need for new biomaterials as ECM.

### Explore the mechanism of drug resistance

To date, multiple mechanisms underlying tumor drug resistance have been identified. At the macro level, these include cancer stem cells^[77]^, immunosuppressive cell subsets^[78]^, angiogenesis^[79]^, and more. At the cellular level, intrinsic changes involve metabolic adaptation^[43]^, defects in apoptosis, inhibition of senescence, autophagy, cellular plasticity, regulation of key targets/signaling pathways, DNA damage response, gene fusions, and interference by miRNAs/lncRNAs. Drug-related mechanisms include reduced drug uptake, increased efflux, metabolic alterations, and segregation of drug-target proteins^[78]^.

In recent years, researchers have utilized tumor organoids to investigate these resistance mechanisms, typically through a three-step process: (1) Establishing drug-resistant tumor organoids; (2) Comparing normal and drug-resistant organoids to identify key targets and pathways and form hypotheses on resistance mechanisms; (3) Applying molecular biology techniques to manipulate organoids and experimentally validate these hypotheses.

#### Post-construction

Following the construction of both normal and drug-resistant tumor organoids - either derived directly from drug-resistant tumor tissues or induced from normal tumor organoids (step one), and subsequently modified using molecular biological techniques (step three), it is crucial to verify their morphology, DNA integrity, and proliferative capacity. Commonly used assays include:

(1) Hematoxylin and Eosin staining (HE) staining^[80]^ and organoid imaging^[60]^ for morphology observation. (2) Sphere/tube formation assays^[56,73]^ and colony formation assays^[53,54,81]^ to evaluate organoid morphology and quantity. (3) Cyst and sphere analysis (particularly relevant for bone metastatic prostate cancer)^[32]^. (4) Measurement of surface area measurement and identification of organoid types^[60]^. (5) Cell cycle analysis using tools such as lentivirus dual fluorescent markers^[32]^ and Fucci2bl cell cycle indicators^[81]^. (6) Senescence assays to detect cell aging^[43]^. (7) Invasion assays to assess the invasive potential of the cells^[51,54,81]^.

Additionally, PDO-based orthotopic tumor xenograft models in mice are employed, enabling the reconstruction of tumor shape and volume (e.g., for pancreatic cancer) using 3D ultrasound imaging and post-dissection weight measurement^[81]^.

#### Hypothesis formulation

This step involves analyzing gene expression and tumor metabolism using various methods. Genomic sequencing, primarily based on next-generation sequencing (NGS), is the most commonly employed approach^[80]^. Other methods such as targeted DNA sequencing and mitochondrial genome sequencing are currently not widely adopted. Transcriptome analysis methods include RNA-Seq^[56]^, DNA microarray^[54]^, RNA pull-down^[56]^, RNA immunoprecipitation (RIP)^[56]^, and qRT-PCR^[43,81]^. Regarding molecular interactions, FRET-FLIM can be used to study protein-protein/DNA interactions^[43]^. Luciferase can be used to detect the interaction between transcription factors and DNA in the promoter region of target genes^[43,55,56]^. The sequencing methods available for proteomics are even more diverse^[53,56,60,81]^. After data collection, bioinformatics analysis^[55]^, including mutation analysis^[60,81]^, is crucial for processing and comparing the data to identify changes, providing a solid foundation for hypotheses on drug resistance mechanisms.

#### Hypothesis verification

In the third step, researchers conduct hypothesis-driven experiments to address drug resistance. In cases where specific genes are implicated, CRISPR-Cas9 and other genetic engineering techniques are used to knock out these genes^[55,56]^, or overexpress them either through viral infection^[43,55,56]^ or transient transfection^[81]^. Direct inhibition of key targets is also explored by adding specific inhibitors to see if drug resistance is reversed. For hypotheses related to signaling pathways, such as the p53-B4GALT1-CDK11p110 axis, researchers use genetic engineering to intervene at multiple points in the pathway, including overexpression of upstream regulators P65, generation of stable expression or knockdown models for key proteins, and construction of glycosylation-deficient mutants to study pathway dynamics. Cycloheximide chase experiments further probe these pathways^[81]^.

The technical means used in the study of tumor drug resistance mechanism and the experimental conclusions are summarized in Table 4, revealing an overlap in techniques used before and after hypothesis formulation. Researchers select a combination of techniques to efficiently characterize drug resistance models. Transcriptome and proteome technologies are ubiquitous in research, with qRT-PCR, Western Blot (WB), immunohistochemistry (IF), and immunofluorescence (IHC) being particularly popular. Genome sequencing, such as NGS or WGS, is less frequently used. Specific techniques are often employed for targeted research purposes, particularly when the direction of resistance mechanisms is known, enabling focused data collection and mechanism exploration.

**Table 4:** Technical means used in the study of tumor drug resistance mechanism and experimental conclusions

**Tumor**		**Lung cancer**	**Pancreatic cancer**				**Hepatocellular carcinoma**		**ESCC and CC^a^**	**Gastric cancer**			**Prostate cancer**	**Colorectal cancer**	**Ovarian cancer**
Drug/treatment		Osimertinib	Gemcitabine	FOLFIRINOX	Gemcitabine/ 5-FU	Oxaliplatin/ paclitaxel/ 5-FU	Sorafenib	Sorafenib	5-FU	Oxaliplatin	Cibisatamab- bound T cells	5-FU	Androgen- targeted therapy	Oxaliplatin	Cisplatin
Mechanism		Tissue type transformation	PRKRA activates the NF-kB pathway	Key signaling pathways are activated	B4GALT1 upregulates N-linked glycosylation of CDK11p110	KRAS-related signaling pathway/ Cell cycle change	BBOX1-AS1/ miR-361-3p/ PHF8 axis	High CD44 expression/ Hedgehog signaling	High CD44 expression/ autophagy	High MYOF expression	Existence of CEA^-/lo^ cells	High KHDRBS3 expression	Dormant tumor cells with basal-luminal phenotype	Lnc-RP11-536 K7.3/SOX2/HIF-1α signaling axis	Aurora-A/ SOX8/ FOXK1 signaling axis/ glucose metabolism induction
Reference		[57]	[55]	[60]	[81]	[61]	[62]	[51]	[33]	[54]	[82]	[53]	[32]	[56]	[43]
Genomics	NGS^b^	√				√									
	Mutation analysis			√		√									
Transcriptomics	RNA-seq				√	√							√	√	√
	qRT-PCR		√	√	√		√	√		√	√	√	√	√	√
	DNA Microarray									√		√			
	RIP^c^						√							√	
	RNA pull-down													√	
	Luciferase reporter assay		√		√		√							√	√
Proteomics	WB^d^		√	√	√		√	√		√		√		√	√
	IP^e^													√	√
	Co-IP^f^				√										
	ChIP^g^		√		√										√
	Mass Spectrometry													√	√
	Proteome profiler array			√											
	Subcellular fractionation														√
	Flow cytometry			√					√		√				√
	Cycloheximide chase assay				√										
Drug response evaluation	Drug sensitivity testing	√	√	√	√	√	√	√	√	√	√	√		√	√
Bioinformatics	Other bioinformatic analyses		√										√		
Functional validation	Gene knockout/ knockdown		√		√		√			√		√		√	
	Gene overexpression		√		√		√			√		√		√	√
	Xenograft		√		√		√	√	√	√	√	√		√	√
	Sphere/ tube formation				√			√		√		√		√	
	Colony formation				√			√			√			√	
	Cyst/ spheroid analysis												√		
	Cell invasion		√		√		√	√		√					
	Beta-galactosidase activity														√
Metabolic analysis	Metabolic analysis														√
	Glycolysis analysis													√	√
	Oxygen consumption rate/ extracellular acidification rate														√
Imaging and morphology	IF^h^	√	√				√	√					√	√	√
	IHC^i^	√	√		√			√	√	√		√	√	√	√
	H&E Staining			√											
	Organoid imaging			√		√									
	Surface measurement			√									√		
	Cell cycle imaging												√		
	FRET-FLIM^j^														√

The "√" symbol in the table indicates that the corresponding technical method was used in this study. Where the symbol is absent, the method was not used. ^a^ESCC & OPSCC: Esophageal squamous cell carcinoma & oropharyngeal squamous cell carcinoma; ^b^NGS: next-generation sequencing; ^c^RIP: RNA immunoprecipitation; ^d^WB: western blot; ^e^IP: immunoprecipitation; ^f^Co-IP: co-immunoprecipitation^[55,81]^; ^g^ChIP: chromatin immunoprecipitation; ^h^IF: immunohistochemistry; ^i^IHC: immunofluorescence; ^j^FRET-FLIM: Fӧrster resonance energy transfer-fluorescence lifetime imaging.

Of course, not all drug resistance studies follow a set pattern. Before organoid experiments, some teams conduct pathological and molecular tests on the patient to analyze transcriptomic/genomic differences in cancer cells with varying drug sensitivities^[57]^. For hypothesis testing, certain researchers may use tumor organoids alongside cell lines^[54,55,81]^ or xenotransplantation^[43,53]^.

## OUTLOOK AND CONCLUSION

### Deficiency of modeling

Despite their transformative potential, tumor organoid models face critical challenges. Examples are as follows:

(1) Tumor heterogeneity leads to varying complexities in culturing different organoid types. This heterogeneity hinders the standardization of cancer organoid modeling^[83]^. Therefore, some cancers, such as liver and prostate cancers, have low rates of 15% to 30%^[84,85]^. (2) A single organoid may not represent the entire tumor due to intra-tumor heterogeneity^[67]^, which may lead to varying drug sensitivities among cancer cells from different tumor regions. (3) Most of the reported cancer organoids originate from the epithelium, with only two non-epithelial organoids identified^[45,86]^. Suitable modeling methods for non-epithelial cancer organoids remain to be explored. (4) Most organoids necessitate sourcing primary cells from patient tissues, which can be challenging for research institutions lacking direct access to hospitals. (5) Current culture systems lack *in vivo* components and are not influenced by neurohumoral regulation. Recreating the microenvironment with Cancer-Associated Fibroblasts or capillaries is challenging^[83]^. Due to tumor heterogeneity, slight variations in culture components can lead to genetic and structural differences, making it difficult to predict clinical drug responses. (6) Compared with 2D cell lines, cancer organoids are more difficult to culture and analyze in high-throughput settings because of their longer cultivation cycles, more complex culture conditions, and higher associated costs.

The efficient translation of experimental results to clinical applications is influenced by the power of experimental models. Organoid culture must not only closely mimic the complexity of TME, but also reduce cultivation cycles and modeling difficulty. As technology progresses and demands grow, researchers have achieved significant breakthroughs.

### Progress in simulation

Researchers have advanced TME simulation by co-culturing organoids with fibroblasts, immune cells^[8]^, and even incorporating DNA microarrays^[74,75]^. Techniques such as the air-liquid interface (ALI)^[87,88]^ and the use of new ECMs^[83]^ have further improved cultivation systems.

The ALI method preserves the tumor’s native immune and stromal cells, and cytokines such as IL-2 help maintain immune cell activity^[87]^. This method has enabled the successful simulation of immune checkpoint blockade and the cancer immune cycle, allowing organoid models to be used for studying drug effects on immune cell regulation in the TME^[88]^.

New biosynthetic ECMs are non-toxic, biocompatible, and customizable, enabling the incorporation of specific TME cell components for co-culture^[83]^. ECMs are generally categorized into natural hydrogels, synthetic hydrogels^[89]^, and non-hydrogel matrices. Natural hydrogels, which can be modified with ECM motifs such as RGD peptides and protease-degradable sequences, are commonly used for culturing intestinal organoids^[90]^. Non-hydrogel matrices use degradable polymers as porous scaffolds, facilitating nutrient exchange and growth control, making them suitable for culturing organoids derived from bone tumors or bone metastases^[71]^.

### Progress in efficiency

Efforts to boost organoid culture efficiency are focusing on developing automated microfluidic platforms^[91,92]^, applying AI^[93]^, and creating organoid biobanks.

Automated microfluidic platforms for organoid culture integrate organoids with microfluidic technology to seed a statistically representative density of heterogeneous cell populations from parental tumor tissues into microwells^[94]^. They allow for the computer-controlled addition of growth factors at regular intervals, enabling high-throughput cultivation and dynamic regulation of organoids^[95]^. This approach shortens modeling time, decreases labor costs, and minimizes batch-to-batch variability in organoid shape, size, composition, and gene expression.

AI is pivotal for big data analysis and understanding variable relationships. AI’s image recognition capabilities, particularly deep learning, enable the analysis of organoid growth changes in shape and size. For instance, OrganoID^[93]^ can automatically identify, label, and track organoids with high accuracy, closely matching manual methods for counting (95%) and size measurement (97%).

The first tumor organoid bank^[96]^, established in 2015, has led to the creation of various tumor organoid libraries. These biobanks allow for the efficient preservation and management of diverse organoids, enabling quick access when needed, saving time and costs for researchers.

### Future directions

In drug resistance research, exploring treatment options and causes of resistance are key, complementary areas that drive progress. Organoid-based drug screening helps in precision medicine, reducing tumor recurrence and resistance, while insights into resistance mechanisms can guide drug screening and offer new perspectives.

As research progresses, more techniques will likely be applied to tumor evaluation. Future optimizations could involve developing new assays to enhance detection scope, efficiency, and accuracy, or tailoring assay combinations for comprehensive information at lower costs and in less time. Currently, organoid detection and analysis largely mirror 2D cell line experiments, which do not fully leverage the advantages of 3D organoids growing in matrix gels^[32]^. Some more suitable techniques^[97]^ are underutilized due to high costs, a limitation that future research must address.

Recent literature indicates that breakthroughs in tumor organoid research are driven by interdisciplinary collaboration among biomedicine, materials science, and engineering. Tumor organoids are integrated with various technologies in drug resistance research, including Organoids-on-a-chip^[9,76]^, single-cell and high-throughput technologies, CRISPR-Cas9 transgene therapy, 3D bioprinting, artificial intelligence, and advanced imaging techniques such as CT and PET^[15,74]^. The integration of multidisciplinary methods paved the way for more mature culture techniques and drug resistance studies. Future progress in this field will likely depend on cultivating interdisciplinary expertise and further integrating various disciplines, leading to an “organoid +X” research model that boosts the efficiency of cancer research.

Tumor organoids are transitioning from exploratory tools to central players in cancer research. Despite current limitations, as research and technology advance, organoids are expected to bridge the gap between *in vitro* and *in vivo* studies. By embracing interdisciplinary innovation and focusing on mechanistic depth, they hold unparalleled potential to decode resistance, accelerate drug discovery, and ultimately deliver patient-tailored therapies.

## DECLARATIONS

### Copyright

© The Author(s) 2025.
