# Supplementary material for: Modeling methods of different tumor organoids and their application in tumor drug resistance research
Source: Cancer Drug Resist. 2025 Jul 1;8:32. doi: 10.20517/cdr.2025.34 (PMC12366422; doi:10.20517/cdr.2025.34)
Supplement: Supplementary file 1 [file cdr-8-32-SupplementaryMaterials.pdf]

Supplementary Materials

Modeling methods of different tumor organoids and their application in tumor drug resistance research

Chengming Yang[1], Lushan Yang[1], Yuchen Feng[1], Xingyi Song[1], Shu Bai[1], Sheng Zhang[2], Mingjuan Sun[3]

[1]Department of Student Team, College of Basic Medical Sciences, Naval Medical University, Shanghai 200433, China.
[2]Medical Oncology, Shanghai Cancer Center, Fudan University, Shanghai 200032, China.
[3]Department of Biochemistry and Molecular Biology, Naval Medical University, Shanghai 200433, China.

Correspondence to: Prof. Sheng Zhang, Medical Oncology, Shanghai Cancer Center, Fudan University, No.270 Dong'an Road, Shanghai 200032, China. E-mail: wozhangsheng@hotmail.com; Dr. Mingjuan Sun, Department of Biochemistry and Molecular Biology, Naval Medical University, No.800 Xiangyin Road, Shanghai 200433, China. E-mail: sunmj@smmu.edu.cn

Preparation

1 Thaw ECM in the refrigerator or freezer at 4°C for at least 12 hours before use. If necessary, dispense it into 1 ml aliquots in 1.5 ml tubes to avoid freeze-thaw cycles.

2 Sterilize all the equipment required for the culture.

3 Place sterilized pipette tips in a refrigerator or freezer at 4°C to prevent premature polymerization of ECM and reduce the coating of ECM on the surface of tips when handling ECM.

4 Configure reagents such as complete organoid culture medium and the mixture of ECM and organoid culture medium.

5 Preheat the medium, culture plates, and PBS

Establishment of organoid from primary tumor tissue or cells

1 Obtain required tumor samples by suitable means.

Tip: At this point, one or two representative fragments can be fixed in a fixative (i.e., 10% formalin or 4% paraformaldehyde) for histological analysis, rapidly frozen fragments can be used for molecular (i.e., whole-exome/genome sequencing or mRNA sequencing) or biochemical (i.e., Western blotting or proteomics) analysis at -80°C. These fragments can also be used to isolate DNA by SNP/STR fingerprinting for quality control or verification of patient origin.

2 The appropriate size of cell mass is precipitated by mechanical disruption such as pipetting, enzymatic digestion, filtration through a cell strainer, and centrifugation. Cells from part of the sampling route do not have to go through some of these steps, as will be mentioned in Section 1.1.2.

3 resuspend the precipitate in working medium, determine the cell density and calculate the number of cells and the amount of medium and ECM required.

4 Further adjust the cell density by dilution or resuspension in the medium after centrifugation

5 Mix the pellets with ECM or resuspend them directly in the medium -ECM mixture after centrifugation.

Tip:

1 The ECM should be kept on ice and the mixing action should be rapid to prevent solidification.

2 The amount of ECM depends on the number of cells, approximately 10,000 cells were

dispensed in 40µl of BME.

2 To ensure solid droplet formation, The proportion of BME should be >70%, Although a lower ratio such as 60% is also mentioned in some literatures, do not dilute BME too much.

6 Drop the medium-ECM mixture containing the organoids on the bottom of the pre-warmed plate wells.

Tip:

1 Plates should be preheated overnight in a humidified incubator at 37°C. If they are not properly preheated, they can be heated at 60°C for 1 hour and then incubated at 37°C for 30 min, otherwise droplets will immediately scatter and flatten on the surface of the plate, and the organoids will easily attach to the bottom of the plate.

2 After resuspension, dispensing should be performed as soon as possible, otherwise the hydrogel may solidify in the tube or pipette tip.

3 If neighboring droplets fuse with each other, the droplet volume can be reduced or the droplet spacing can be increased. Occasional droplet fusion does not have a negative effect on organoid growth.

7 Incubate the plates in an incubator at humidified, 37°C, 5% (vol/vol) CO_2 to solidify the ECM.

8 After the droplets had solidified, take out the plates and carefully add pre-warmed organoid medium to each well.

Tip: Do not add media directly to the BME droplet, this will most likely destroy the droplet.

9 Place the plates in an incubator at humidified,37°C and 5% (vol/vol) CO_2. Change the medium every 2-3 days: carefully aspirate the medium from the wells and replace it with fresh, pre-warmed medium.

Tip:

1 BME droplets may detach from the bottom of the plate and 'swim' in the medium; in this case, it is recommended to re-plate the organoids without the use of TrypLE and closely monitor the status of organoid formation.

2 The insufficient dilution of the trypsin used for digestion or the protease released by

the primary cells may lead to the digestion of ECM overnight. If so, wash the plate with medium, replate pellet in fresh ECM and spread it into a new plate with medium containing caspofungin, Primocin and ROCK inhibitors after solidification.

Organoid passage

Passages were performed when the organoids exhibited vigorous growth and sufficient BTM supply, or when there was a threat to the integrity of the dome, as well as when light microscopy indicated that membrane vesicles were releasing signals of impending apoptosis.

Tip:

1 Do not passage too quickly, as this may result in growth termination; ideally, the first passages of the organoids should be made between 12 and 14 days after the initial laying of the plates.

2 It is recommended that ROCK inhibitors and Primocin, and caspofungin if head and neck organoids, be maintained in the medium for the first two passages of cultivation.

1 Blow to break up the gel and transfer the organoid suspension to a 15 ml conical tube that is placed on ice. Add 10 ml of ice-cold medium and blow to help dissolve and remove the gel. dispase can also be added for digestion of ECM.

2 Centrifuge the organoid at 4°C and aspirate the supernatant.

3 Divide the organoids by either TrypLE digestion or mechanical disruption. If using enzymatic hydrolysis, resuspend the precipitate in TrypLE and blow appropriately, then incubate it at 37°C and monitor digestion closely until the organoids are digested to the desired state. If using mechanical disruption, resuspend the precipitate in the medium and blow on the suspension of organoids with the tip of the pipette. Blowing into the bottom of the tube can create higher pressure that aids in the disruption of the organoids.

Tip:

1 Do not digest in TrypLE for too short time or more than 15 minutes: over-digestion may result in poor or even cessation of organoid growth; while lack of digestion may lead to the removal of large cell clumps or organoid fragments during the filtration, causing small yields of cells when collected. As a rule of thumb, the digestion is completed when a mixed cell mass that consisting of 2-10 cells can be observed.

2 If the organoid keratinizes while growing, making it difficult to destroy the structure,

the tip of the gun can be over-fired to make the opening smaller. As a result, the destruction efficiency can be improved.

4 Filter the organoid suspensions using cell strainer.

5 Consistent with steps 3-8 of primary modeling.

Cryopreservation and thawing of organoids

1 2-3 days after the passage, obtain organoid precipitates according to the steps 1 and 2 of organoid passage.

Tip: To ensure that the gel can be removed by washing, a maximum of 12 well contents of a 24-well cell culture plate can be assembled in a 15ml conical tube.

2 Add the desired volume of cell freezing medium and blow with a pipette to resuspend the organoid properly.

3 Place the cryovial in the freezer container. And the freezer container containing the samples was stored at -80°C freezer.

4 After storage at -80 ° C for 24 h, the samples were removed from the freezer container and transferred to a liquid nitrogen tank (approximately -180 °C).

5 Prepare a 15 ml centrifuge tube for each frozen tube to be thawed and add 10 ml of culture medium at room temperature.

6 Remove the cryotubes containing organoids from the liquid nitrogen tank and thaw the frozen medium by incubating the cryopreserved tubes in a 37°C water bath.

Tip: The thawing should be carefully monitored, and the cryovial should be removed from the water bath as soon as the freezing medium has thawed; given the time required to walk to the incubator, vials can be removed from the water bath when a small cloud of ice is present.

7 Add a small amount of medium into the freezing tube, then transfer all the liquid in the freezing tube to a 15 ml conical tube.

8 Centrifuge the organoid at 4°C and aspirate the supernatant.

9 Consistent with steps 3-8 of primary modeling.

Tip: If the freezing medium is not diluted at least 10-fold during the washing process, additional washing steps should be performed to ensure that the freezing medium is adequately diluted before plating.
